# eHealth Literacy and Depressive Symptoms Among Adults Aged 50 Years and Older in China: A National Cross‐Sectional Study

**DOI:** 10.1155/da/3142110

**Published:** 2026-10-28

**Authors:** Qin Zhou, Shengpeng Guo, Rize Jing

**Affiliations:** ^1^ School of Public Administration and Policy Renmin University of China Beijing China ruc.edu.cn; ^2^ Institute for Global Health and Development Peking University Beijing China pku.edu.cn; ^3^ Bigdata and Responsible Artificial Intelligence for National Governance Renmin University of China Beijing China ruc.edu.cn; ^4^ Zhengzhou Police University Zhengzhou China; ^5^ Center for Population and Development Studies Beijing Higher Education Innovation Center for Population Development and Governance Research Renmin University of China Beijing China ruc.edu.cn; ^6^ School of Population and Health Renmin University of China Beijing China ruc.edu.cn

**Keywords:** adults aged 50 years and older, depressive symptoms, digital health, eHealth literacy, perceived stress, physical activity

## Abstract

**Objective:**

Despite existing research confirming that eHealth literacy promotes healthy lifestyles and subjective well‐being among adults aged 50 years and older, its connection to depressive symptoms remains unclear. This cross‐sectional study explored the association between eHealth literacy and depressive symptoms among adults aged 50 years and older, and investigated potential mechanisms and moderating effects of the association.

**Methods:**

Data were obtained from the China Digital Divide and Digital Inclusion Survey (CDDDIS), which provided a nationally representative sample of 4284 participants aged 50 years and older. Ordinary least squares (OLS) regression was employed to analyze the associations between eHealth literacy and depressive symptoms, adjusting for relevant sociodemographic and health‐related covariates. Bootstrap‐based mediation analysis was conducted to evaluate indirect pathways embedded within the correlation network. Income‐stratified analyses were performed to explore differences in the association across income groups.

**Results:**

The results revealed that higher eHealth literacy was significantly correlated with fewer depressive symptoms (*β* = −0.029, 95% CI = [−0.052, −0.006], *p* = 0.012). Bootstrapped mediation analysis revealed that physical activity and perceived stress served as significant indirect pathways mediating the association in this correlational analysis, whereas the indirect pathway via online healthcare services utilization was not statistically significant. Income‐stratified analysis showed that the negative association between eHealth literacy and depressive symptoms was statistically significant among middle‐ and high‐income participants, whereas no significant association was observed for low‐income participants.

**Conclusions:**

eHealth literacy constitutes a critical resource for promoting healthy aging and mitigating aging‐related stressors among adults aged 50 years and older. These correlational patterns suggest potential utility for targeted eHealth interventions aimed at reducing depressive symptoms in aging populations.

## 1. Introduction

The World Health Organization defines healthy aging as the process of developing and maintaining the functional ability that enables well‐being in older age [1]. Mental health and psychological well‐being are integral to this process. Depressive disorders constitute an important public health concern in China, with their lifetime prevalence among Chinese adults estimated at 6.8% [2]. Although depressive symptoms identified using screening instruments are not equivalent to a clinical diagnosis of depressive disorder, they are associated with impaired daily functioning, poorer quality of life, increased healthcare utilization, and elevated mortality risk [3]. Identifying factors associated with depressive symptoms is therefore important for promoting psychological well‐being and healthy aging among adults in later middle and older adulthood.

The growing use of digital technologies has expanded access to health information and mental health support. eHealth literacy refers to the capacity to seek, understand, evaluate, and apply health information using digital devices and online resources [4]. It may be particularly relevant to adults in later middle and older adulthood, who may have increasing health‐information needs while encountering barriers to the effective use of digital health resources [5]. In China, the rapid growth of internet use among older adults has made online platforms an increasingly important channel for accessing health information and medical services. By December 2022, there were 153 million internet users aged 60 years and above, with a penetration rate exceeding 50% [6]. This trend has drawn increasing research attention to the relationship between eHealth literacy and health‐related outcomes in later life [7].

Higher eHealth literacy has been associated with more active health‐information‐seeking and health‐promoting behaviors [8], as well as greater life satisfaction and subjective well‐being [9]. Preliminary evidence also suggests that higher eHealth literacy is linked to fewer symptoms of anxiety and depression [10]. However, most existing studies have focused on working populations, patients with specific clinical conditions, or university students [11, 12], with relatively limited attention to adults in later middle‐ and older adulthood. Differences in study populations, measurements, and analytical approaches also constrain the generalizability of the previous findings.

Important questions concerning this association remain insufficiently addressed. In particular, self‐reported physical activity frequency, perceived stress, and online healthcare service utilization may constitute statistical indirect pathways between eHealth literacy and depressive symptoms, but these pathways have rarely been examined simultaneously. Among these potential pathways, perceived stress has a particularly clear theoretical basis. Stress and coping theory emphasizes the relevance of individuals’ appraisal of stressors and perceived coping resources [13], whereas cognitive vulnerability theories of depression highlight the associations of persistent negative appraisal, reduced perceived control, and maladaptive cognitive patterns with depressive symptoms [14]. Together, these perspectives provide a conceptual basis for examining perceived stress as a potential statistical indirect pathway between eHealth literacy and depressive symptoms. Moreover, possible variation in this association across income groups has received limited attention. Because income may shape access to digital devices, health information, and healthcare resources, examining income‐stratified patterns may provide a more nuanced understanding of this association.

To address these gaps, we analyzed data from a large national survey of adults aged 50 years and older with broad geographic coverage across China. This study aimed to examine the association between eHealth literacy and depressive symptoms and to assess potential statistical indirect pathways through self‐reported physical activity frequency, perceived stress, and online healthcare service utilization. We hypothesized that higher eHealth literacy would be associated with fewer depressive symptoms and that statistically significant indirect associations would be observed through these three variables. We also explored whether the association differed across income groups. By simultaneously examining the primary association, multiple statistical indirect pathways, and income‐stratified patterns, this study adds new evidence on the relationship between eHealth literacy and depressive symptoms among adults aged 50 years and older in China.

## 2. Theoretical Basis and Hypotheses Development

### 2.1. eHealth Literacy and Depressive Symptoms

The stress and coping theory provides a theoretical framework for examining psychological stress responses [15]. It is not proposed as a theory explaining the etiology of depression, but rather as a process‐oriented framework for understanding how eHealth literacy may be associated with depressive symptoms through differences in stress appraisal and coping resources. According to this theory, stress is context‐dependent, arising from dynamic interactions between individuals and their environments, and process‐oriented, evolving over time [16]. Middle‐aged and older adults frequently encounter multiple stressors, including age‐related physiological decline and psychological adjustment challenges, which may significantly threaten their physical and mental well‐being [17]. Among these stressors, depressive disorders represent a prevalent and serious mental health concern.

With the increasing integration of digital technologies into healthcare, various stress management strategies have emerged, with enhancing eHealth literacy being recognized as an effective coping mechanism [18]. Grounded in the stress and coping theory, higher eHealth literacy may be associated with more adaptive appraisal and coping patterns, which may, in turn, be linked to fewer depressive symptoms among middle‐aged and older adults. Specifically, eHealth literacy may be associated with a more structured appraisal process, enabling individuals to assess the potential health impact of stressors (primary appraisal) and evaluate their available coping resources and self‐efficacy (secondary appraisal). This dual appraisal process may support a more accurate assessment of stressor severity and identify viable coping strategies.

Beyond appraisal, eHealth literacy may be associated with greater use of coping strategies in response to stressors. For example, when faced with health‐related stressors, individuals with high eHealth literacy may use online resources to access professional healthcare and medical advice or solutions [10]. Empirical research indicates that patients with higher eHealth literacy demonstrate superior health information‐seeking skills and are more likely to obtain reliable, high‐quality health information compared to those with limited eHealth literacy, factors that contribute to improved health outcomes [19].

In addition, socioeconomic conditions, particularly income, may shape the context in which the appraisal and coping processes unfold. According to the stress and coping theory, secondary appraisal is influenced not only by personal capabilities but also by tangible resources such as income [15]. Individuals with higher income tend to have better access to digital infrastructure and perceive greater coping capacity, which may amplify the inverse association between eHealth literacy and perceived stress [11]. Conversely, low‐income older adults face material constraints and competing demands (e.g., financial strain and caregiving burden) that may attenuate the inverse association between eHealth literacy and depressive symptoms. Therefore, we further examine whether this association varies across income levels in our empirical analysis.

**Hypothesis 1.**

eHealth literacy is negatively associated with depressive symptoms among adults aged 50 years and older.

### 2.2. Theoretical Analysis of Potential Indirect Pathways

#### 2.2.1. Rationale for Mediator Selection

The selection of the three mediating variables (e.g., online healthcare service utilization, physical activity, and perceived stress) was guided by a combination of theoretical relevance, empirical evidence, and data availability. From a theoretical standpoint, the stress and coping theory distinguishes between problem‐focused coping (directly addressing the stressor) and emotion‐focused coping (regulating emotional responses) [20]. Online healthcare service utilization and physical activity represent two complementary problem‐focused coping strategies (one digital and one behavioral), while perceived stress is included as a cognitive‐emotional mediator reflecting the appraisal process.

Empirically, these variables have been identified in prior studies as key pathways linking health literacy to mental health outcomes [21–23]. Other potential mediators (e.g., health information seeking, social support, and medication adherence) were considered but were not comprehensively measured in the survey. Data availability thus constrained the scope of our mediation analysis.

#### 2.2.2. Potential Problem‐Focused Coping Pathways: Online Healthcare Services Utilization and Physical Activity

As physical function deteriorates and the risk of chronic diseases increases among adults aged 50 years and older, their demand for medical, health, and nursing services gradually rises. In this context, eHealth literacy plays a critical role in enabling individuals to efficiently seek, evaluate, and utilize relevant digital health resources when managing health‐related challenges [24]. Empirical evidence suggests that higher eHealth literacy is associated with increased engagement in online healthcare services, a key problem‐focused coping strategy [25, 26]. For example, individuals with greater eHealth literacy are more likely to schedule doctor appointments, seek professional health consultations online, and obtain information about health support groups online. These actions may be associated with greater self‐efficacy and perceived control in dealing with health‐related stressors [27].

Moreover, the utilization of online healthcare service may be associated with fewer depressive symptoms, potentially through improved access to healthcare, particularly for older adults who face mobility constraints or live in underserved areas [28]. Timely access to professional health information and virtual consultations may be associated with less health‐related uncertainty and greater perceived control over their health conditions and mitigate the psychological distress associated with unmet medical needs [29, 30]. Conversely, delayed or foregone care due to access barriers has been linked to elevated depressive symptoms in older populations [31, 32]. Thus, by facilitating timely and convenient healthcare access, online healthcare service utilization may serve as an effective problem‐focused coping strategy that not only addresses health concerns but also may be associated with a lower emotional burden related to untreated or poorly managed conditions.

Thus, higher eHealth literacy is associated with greater online healthcare service utilization, functioning as an essential digital pathway for problem‐focused coping, which may constitute an indirect pathway linking eHealth literacy to depressive symptoms in adults aged 50 years and older.

**Hypothesis 2.**

Online healthcare service utilization constitutes a statistical indirect pathway in the association between eHealth literacy and depressive symptoms.

Physical activity, as a form of healthy lifestyles, is an important protective factor for healthy aging. Higher eHealth literacy may be associated with more favorable secondary appraisals of adults aged 50 years and older regarding the necessity and importance of adhering to healthy lifestyles, such as engaging in physical exercise [33]. They can fully recognize the benefits that regular physical exercise, a balanced diet, and other healthy behaviors bring to maintaining their activities of daily living, delaying declines in cognitive and cardiopulmonary functions, reduced risk of chronic disease development, and improved chronic disease management outcomes [34].

Empirical research has established eHealth literacy as a significant determinant in both the adoption and maintenance of healthy lifestyles practices [35]. Furthermore, studies indicate that eHealth literacy has been positively associated with health status and health‐promoting behaviors [36, 37]. In this context, physical activity can be seen as an important offline channel of problem‐focused coping strategies, enabling individuals to actively mitigate age‐related health threats. Consequently, higher eHealth literacy may be associated with more frequent engagement in physical activity. By doing so, they can maintain physical health, fend off potential risks, enhance self‐efficacy in health management, and develop positive health outcome expectations. These positive outcomes subsequently contribute to the alleviation of depressive symptoms. More frequent physical activity may therefore represent a potential pathway linking eHealth literacy to fewer depressive symptoms.

**Hypothesis 3.**

Physical activity constitutes a statistical indirect pathway in the association between eHealth literacy and depressive symptoms.

#### 2.2.3. Perceived Stress as a Cognitive‐Emotional Mediator

Perceived stress in middle‐aged and older adults constitutes a subjective psychological construct reflecting their cognitive appraisals of challenges encountered during the aging process [38]. In the stress and coping theory, perceived stress is not a coping strategy per se, but rather the cognitive‐emotional outcome of the appraisal process [20]. Stress appraisal involves two stages: primary appraisal (evaluating the significance of a stressor) and secondary appraisal (evaluating one’s coping resources and options). Perceived stress reflects the subjective experience that environmental demands exceed available coping capacities [39].

Higher eHealth literacy may be associated with lower perceived stress through differences in appraisal processes and coping resources. First, access to accurate health information among individuals with higher eHealth literacy may be associated with less uncertainty when they encounter health‐related stressors and threat perception when facing health‐related stressor [18]. Second, eHealth literacy strengthens secondary appraisal by providing knowledge of available coping options (e.g., online consultations and lifestyle modifications), thereby enhancing self‐efficacy and perceived control [40]. Through these appraisal mechanisms, higher eHealth literacy may therefore be associated with lower perceived stress levels. In this framework, depressive symptoms are understood as the psychological distress outcome that emerges when stressors are appraised as overwhelming and coping resources are perceived as insufficient [16]. eHealth literacy, by enhancing both appraisal accuracy and coping repertoires, may be linked to differences in this pathway and, consequently, to fewer depressive symptoms.

The link between perceived stress and depressive symptoms is well‐established in the literature. Chronic elevated perceived stress activates the hypothalamic–pituitary–adrenal (HPA) axis, leading to sustained cortisol release, which has been linked to the onset and exacerbation of depressive symptoms [41]. Psychologically, persistent perceptions of being overwhelmed by environmental demands erode self‐efficacy, promote negative cognitive schemas, and foster learned helplessness, all of which are proximal risk factors for depressive disorders [42, 43]. In older adults, the association between perceived stress and depressive symptoms is particularly pronounced, as age‐related declines in physiological resilience and social roles may amplify the psychological impact of stress [23, 44]. Consequently, perceived stress may serve as an indirect pathway linking eHealth literacy to depressive symptoms through both neurobiological and psychological mechanisms.

**Hypothesis 4.**

Perceived stress constitutes a statistical indirect pathway in the association between eHealth literacy and depressive symptoms, such that higher eHealth literacy is associated with lower perceived stress, which is, in turn, associated with fewer depressive symptoms.

## 3. Methods

### 3.1. Data and Sample

This cross‐sectional study utilized data from the China Digital Divide and Digital Inclusion Survey (CDDDIS) conducted by the Center for Population and Development Studies, Renmin University of China. The fieldwork was carried out from December 2022 to February 2023. Eligible participants were adults aged 50 years and older who had resided in mainland China for at least 6 months at the time of the survey. The questionnaire collected information covering sociodemographic characteristics, internet access and usage, eHealth literacy, daily activities, and mental well‐being of this population group. A stratified, multistage sampling approach with probability proportional to size (PPS) was adopted to ensure national representativeness. Sampling strata were determined by city administrative rank, population size, economic development level, urbanization rate, and on‐site implementation feasibility. In total, 18 cities, 53 counties (districts and county‐level cities), 126 townships and sub‐districts, and 252 village and neighborhood committees were selected nationwide. Within each selected grassroots unit, 20 eligible individuals were randomly recruited.

To improve sample representativeness, age, gender, and internet usage quotas were established for sampled cities. All quotas were formulated in accordance with the population distribution of residents aged 50 years and older recorded in the Seventh National Population Census in 2020. A total of 5231 face‐to‐face questionnaires were initially collected. After excluding 947 responses with no eHealth literacy assessment due to non‐internet use, missing basic information, or severe logical inconsistencies, the final analytical sample consisted of 4284 participants. To evaluate sample representativeness, sampling weights were calculated based on the 2020 China Family Panel Studies (CFPS) and data from the Seventh National Population Census. Weighted and unweighted results showed only minor discrepancies, indicating that the sample was nationally representative. Accordingly, all analyses in this study were performed using unweighted data. The survey protocol was reviewed and approved by the Ethics Committee of Renmin University of China before data collection. Under the institution’s ethics review procedures in place at the time, questionnaire‐based social science studies that did not involve the collection of biological specimens or other biomedical materials were not assigned a formal ethics approval number. Accordingly, no formal approval number was issued for this survey. All participants provided written informed consent before participating in the survey, and the data used in the present analysis were anonymized.

### 3.2. Measures

#### 3.2.1. Depressive Symptoms

Depressive symptoms were assessed using the 10‐item short form of the Center for Epidemiologic Studies Depression Scale (CESD‐10) [45]. Participants were asked about the frequency of experiencing depressive symptoms over the past week, including getting annoyed because of trivial matters, having difficulty concentrating when doing things, feeling down in spirits, finding it strenuous to do anything, being full of hope for the future, feeling scared, having poor sleep, being very happy, feeling lonely, and feeling that one could not go on with one’s life. The responses categorized as “rarely or not at all,” “not very often,” “sometimes,” and “most of the time” were, respectively, associated with scores ranging from 0 to 3. After reversed scoring of two positively worded items and summing up the scores of 10 items, the total score ranged from 0 to 30 (Cronbach’s *α* = 0.70; KMO = 0.81).

#### 3.2.2. eHealth Literacy

eHealth literacy was assessed using the eight‐item eHealth Literacy Scale (eHEALS) [46]. The eHEALS is a validated self‐assessment instrument that assesses an individual’s perception of her or his own eHealth‐related skills and knowledge. Specifically, the instrument is designed to provide a general estimate of consumer knowledge of what health information is available on the Internet, where to access useful information, how to search for it online, and how to use it to answer personal health questions and assist oneself. Additionally, it evaluates skills in assessing the quality of online health information, distinguishing between high‐ and low‐quality content, and the confidence to make health‐related decisions using such information. Each item was scored on a 5‐point scale ranging from 1 (“not at all capable”) to 5 (“extremely confident”), producing a total score ranging from 8 to 40, with higher scores indicating greater eHealth literacy (Cronbach’s *α* = 0.83; KMO = 0.88). The continuous eHEALS total score was used for the primary analysis. As a complementary analysis, participants were categorized into low (scores of 8–24), middle (scores of 25–28), and high (scores of 29–40) eHealth literacy groups based on sample‐specific tertile cut‐points. Because of tied scores at the tertile cut‐points, the three groups were not equal in size.

#### 3.2.3. Online Healthcare Services Utilization

Drawing on existing measurement frameworks from prior empirical studies, respondents were evaluated on their awareness and uptake of four core types of online medical services [47, 48]. These services included accessing health knowledge and information online, seeking online disease consultations, engaging in online medical consultations, and completing online appointment registrations or medication (electronic prescriptions). Responses were scored 1 if participants were unaware of the service, 2 if participants were aware but never used it, and 3 if participants had used the service. A composite utilization score (range: 4–12) was calculated by summing responses across all four services, with higher scores indicating greater engagement with digital healthcare services.

#### 3.2.4. Physical Activity

Physical activity was assessed through self‐reported monthly exercise frequency. Participants indicated how often they engaged in physical exercise, including various modalities such as walking, running, ball games, the use of fitness equipment, and other aerobic or resistance exercises. The responses of “never,” “≤1 time/month,” “2–3 times/month,” “1 time/week,” “2–3 times/week,” and “nearly daily” were assigned scores from 1 to 6, respectively. A higher score indicated a superior level of physical activity. Higher scores indicated more frequent self‐reported physical activity. This measure captured exercise frequency but did not assess duration, intensity, energy expenditure, or objectively recorded physical activity.

#### 3.2.5. Perceived Stress

Perceived stress was measured using the 10‐item Perceived Stress Scale (PPS‐10) developed by Cohen et al. [39]. The scale consists of positive and negative parts. The positive part consisted of four items that assessed self‐efficacy and ability to control in daily life, such as whether respondents felt confident in handling personal problems, perceived that events unfold as anticipated, were capable of regulating anger, and felt in command when dealing with daily tasks. Conversely, the negative part also contained six items, which focuses on the experiences of stress and helplessness. These items asked whether participants were distressed from unexpected events, felt a lack of control over significant aspects of their lives, experienced nervousness/being overwhelmed, found themselves unable to cope with responsibilities, became angry about uncontrollable situations, and felt overwhelmed by accumulated tasks. The responses “never,” “occasionally,” “sometimes,” “often,” and “always” were assigned scores from 0 to 4, respectively. After reverse‐scoring the four positive items, the perceived stress ranged from 0 to 40, with higher scores indicating greater perceived stress.

We systematically assessed the psychometric characteristics of the PSS‐10 in the present research sample. Preliminary psychometric screening yielded a Cronbach’s *α* of 0.61 and an overall KMO statistic of 0.77, both of which failed to meet the conventional thresholds of 0.7 and 0.8. Such suboptimal preliminary results may be attributed to the scale’s inherent design of mixing positively and negatively phrased items. In line with previous methodological evidence, the KMO statistic serves only as a preliminary indicator for factor analysis suitability and cannot independently refute a valid latent factor structure. Specifically, scales with mixed‐valence items tend to generate inflated partial correlations across indicators, which mechanically reduce the overall KMO value as a statistical artifact without interfering with the extraction of true common latent factors [49].

Accordingly, we conducted principal component analysis to examine the factor structure of PSS‐10. The results clearly revealed a stable two‐factor structure, which perfectly matched the item valence classification: all four positively worded items loaded on one latent factor, whereas all six negatively worded items loaded on the other latent factor (Table S1). Based on this empirically verified two‐factor division, we further evaluated the internal consistency of each subdimension using McDonald’s *ω* coefficient. Different from Cronbach’s *α*, which relies on the strict tau‐equivalence assumption of identical item loadings that rarely holds in practical measurement scenarios, McDonald’s *ω* provides more reliable composite reliability estimation under the congeneric factor framework and is thus preferable for latent construct assessment [50]. The results showed that the negative‐item subdimension yielded a McDonald’s *ω* of 0.77, and the positive‐item subdimension yielded a McDonald’s *ω* of 0.84. Both values exceeded the widely accepted cutoff of 0.7, demonstrating satisfactory internal consistency for each extracted factor.

Furthermore, subsequent confirmatory factor analysis was performed to validate the construct validity of this two‐factor measurement structure. All core model fit indices, including CFI, TLI, SRMR, and RMSEA, satisfied the standard acceptable criteria (Table S2). Collectively, despite the marginal preliminary Cronbach’s *α* and KMO values, the robust factor structure, acceptable dimensional reliability, and favorable CFA fit results collectively confirm that the PSS‐10 possesses adequate psychometric quality for subsequent statistical analyses in this study.

#### 3.2.6. Covariates

Referring to existing empirical studies on eHealth literacy and depressive symptoms [8, 11, 21, 51], a comprehensive set of confounding variables was selected in the present study to eliminate potential biases and obtain robust estimates regarding the association between eHealth literacy and depressive symptoms. These covariates covered multiple dimensions, including demographic characteristics, socioeconomic status, physical functional health conditions, digital access resources, and family contextual factors, which have been well documented to correlate with both eHealth literacy levels and late‐life depressive symptoms. Control variables included age (in years), gender (male = 0, female = 1), residence (rural = 0, urban = 1), education level, marital status (non‐married = 0, married = 1), activity of daily living (ADL) score, number of surviving children, personal annual income (log‐transformed for normalization), household Internet access (no = 0, yes = 1), smartphone ownership (no = 0, has = 1), parental care responsibilities (no = 0, yes = 1), and empty‐nest status (no = 0, yes = 1). Specifically, ADL score was assessed through 5 domains (dressing, bathing, eating, toileting, and mobility). The responses of “fully independent,” “requires partial assistance,” and “fully dependent” were assigned scores from 1 to 3, respectively, and ADL score ranged from 5 to 15. Higher scores indicate greater functional impairment. Income was defined as the total income of participants in the past year, which included all wage‐related income, pensions, financial support from children or grandchildren, investment and financial management earnings, government subsidies, business operation income, and rental income from properties, land, equipment, et cetera.

### 3.3. Statistical Analyses

All analyses were conducted using Stata 17.0. Statistical analyses were performed using two‐tailed tests with a significance threshold of *α* = 0.05 and 95% confidence intervals. First, descriptive statistics were applied to characterize the study sample. For categorical variables, we reported the frequency and proportion of each category; for continuous variables, the mean and standard deviation were presented.

Second, ordinary least squares (OLS) regression was employed to examine the association between eHealth literacy and depressive symptoms. Third, a series of OLS regression models were constructed to test the potential mediating pathways among the study variables. Three potential mediators were included: online healthcare service utilization, physical activity, and perceived stress. To quantitatively verify the existence and significance of the mediating paths, the Bootstrap method with 5000 repeated resamples was implemented. Multicollinearity diagnosis for all regression models yielded variance inflation factor (VIF) values ranging from 1.32 to 1.34, which were substantially below the conventional threshold of 5.0, indicating no serious multicollinearity issues among all variables.

Fourth, we conducted stratified analyses based on the respondents’ personal annual income to explore heterogeneous associations across different income levels. All participants were divided into low‐, middle‐, and high‐income groups according to the tertiles of personal annual income. OLS regression was run within each subgroup to compare the association between eHealth literacy and depressive symptoms across distinct income brackets. To further examine the moderating role of household income in the correlational linkage between eHealth literacy and depressive symptoms, interaction terms of income level and eHealth literacy were incorporated into the regression model.

Finally, a series of supplementary robustness analyses were performed to validate the stability of the primary findings and mitigate the selection bias within this observational research framework. Propensity score matching (PSM) was further implemented to balance observable pre‐existing characteristics across eHealth literacy subgroups and reduce confounding bias. This method constructs propensity scores to align covariate distributions between the comparison groups. Sample stratification followed the sample mean of eHEALS scores: respondents with eHEALS scores at or above the sample mean were assigned to the high eHealth literacy group, and those with scores below the mean formed the low eHealth literacy group. All covariates incorporated in the baseline regression were included as matching variables to eliminate intergroup disparities. Following the achievement of an adequate covariate balance, propensity score‐derived analytical weights were generated, and weighted OLS regression was performed to re‐examine the relationship between eHealth literacy and depressive symptoms.

As an additional weighting‐based robustness strategy, inverse probability weighting (IPW) was adopted. Consistent with the group division criterion applied in the PSM procedure, participants were classified into high and low eHealth literacy subgroups based on the sample mean of eHEALS. Inverse probability weights were computed from a propensity score logistic model adjusting for the full set of pre‐specified covariates, after which weighted OLS regression was conducted to estimate the average treatment effect of elevated eHealth literacy on depressive symptoms, providing further corroborating evidence for the reliability of the core estimation results.

## 4. Results

### 4.1. Sample Characteristics

Table 1 presents the sample characteristics of the 4284 participants. The participants had a mean age of 59.00 ± 6.96 years, with females making up 50.19% of the sample. A total of 68.93% lived in urban areas. In terms of education, 22.50% had primary school education or below, 34.48% had middle school education, 34.80% had a high school degree, and 8.22% held a college degree or higher. Most participants (92.83%) were married, 5.09% were empty nesters, and 19.05% undertook parental care work. The mean number of surviving children was 1.53 ± 0.85. In total, 95.17% of households had internet access, and 98.81% of participants owned a smartphone. The mean score of activities of daily living was 5.11 ± 0.53, and the average income score was 1.89 ± 0.62. The mean score of depressive symptoms was 7.52 ± 3.99. The overall mean eHealth literacy score was 26.34 ± 5.57. The mean scores of online healthcare service utilization, physical activity, and perceived stress were 8.00 ± 1.60, 4.70 ± 1.38, and 14.60 ± 4.61, respectively.

**Table 1:** Sample characteristics (*N* = 4284).

Variables	Mean ± SD/*N* (%)
Age (range: 50–89)	59.00 ± 6.96
Gender	
Female	2150 (50.19)
Male	2134 (49.81)
Residence	
Rural	1331 (31.07)
Urban	2953 (68.93)
Education level	
Primary school and below	964 (22.50)
Middle school	1477 (34.48)
High school	1491 (34.80)
College and above	352 (8.22)
Marital status	
Yes	3977 (92.83)
No	307 (7.17)
Activity of daily living (range: 5–15)	5.11 ± 0.53
Household Internet access	
Yes	4077 (95.17)
No	207 (4.83)
Smartphone ownership	
Yes	4233 (98.81)
No	51 (1.19)
Surviving children number (range: 0–14)	1.53 ± 0.85
Parental care responsibilities	
Yes	816 (19.05)
No	3468 (80.95)
Empty‐nest status	
Yes	218 (5.09)
No	4066 (94.91)
Income (range: 0–3.93)	1.89 ± 0.62
Depressive symptoms	7.52 ± 3.99
eHealth literacy (range: 8–40)	26.34 ± 5.57
Online healthcare services utilization (range: 4–12)	8.00 ± 1.60
Physical activity (range: 1–6)	4.70 ± 1.38
Perceived stress (range: 0–40)	14.60 ± 4.61

### 4.2. The Association Between eHealth Literacy and Depressive Symptoms

Higher eHealth literacy was significantly correlated with fewer depressive symptoms (*β* = −0.029, 95% CI = [−0.052, −0.006], *p* = 0.012) (Table 2). Based on tertile cut‐points, participants were classified into low (*n* = 1684, 39.31%), middle (*n* = 1467, 34.24%), and high eHealth literacy groups. When eHealth literacy was stratified into tertiles, participants in the middle (*β* = −0.345, 95% CI = [−0.634, −0.055], *p* = 0.020) and high literacy groups exhibited lower levels of depressive symptoms relative to those in the low literacy group (Table S3). Thus, Hypothesis H1 was supported.

**Table 2:** Ordinary least squares regression between eHealth literacy and depressive symptoms.

Variables	*β*	95% CI	*p*
eHealth literacy	−0.029	[−0.052, −0.006]	0.012
Age	0.026	[0.005, 0.047]	0.015
Female	0.480	[0.244, 0.717]	<0.001
Residence (ref: rural)	−0.681	[−0.942, −0.420]	<0.001
Education level (ref: primary school and below)			
Middle school	−0.042	[−0.368, 0.284]	0.802
High school	0.010	[−0.317, 0.338]	0.950
College and above	−0.183	[−0.677, 0.311]	0.467
Marital status (ref: no)	−1.012	[−1.706, −0.318]	0.004
Activity of daily living	0.902	[0.633, 1.172]	<0.001
Household Internet access (ref: no)	−1.023	[−1.543, −0.503]	<0.001
Smartphone ownership (ref: no)	−0.629	[−1.762, 0.504]	0.276
Number of surviving children	0.326	[0.147, 0.504]	<0.001
Parental care responsibilities (ref: no)	0.203	[−0.106, 0.511]	0.197
Empty‐nest status (ref: no)	0.547	[−0.254, 1.347]	0.181
Log (income)	0.380	[0.150, 0.611]	0.001
Constant	3.657	[1.067, 6.248]	0.006

*Note: N* = 4284, *R* ^2^ = 0.049.

Abbreviations: CI, confidence interval; Ref, reference.

### 4.3. Statistical Indirect Pathways Through Online Healthcare Services Utilization, Self‐Reported Physical Activity Frequency, and Perceived Stress

Correlational pathways are presented in Figure 1 and Tables S4 and S5. Adjusted estimates showed eHealth literacy was positively associated with online healthcare services utilization (*β* = 0.096, 95% CI = [0.087, 0.106], *p* < 0.001) and reported physical activity frequency (*β* = 0.043, 95% CI = [0.035, 0.052], *p* < 0.001) and negatively associated with perceived stress (*β* = −0.036, 95% CI = [−0.062, −0.011], *p* = 0.005). Physical activity was negatively associated with depressive symptoms (*β* = −0.241, 95% CI = [−0.325, −0.157], *p* < 0.001), while perceived stress was positively associated with depressive symptoms (*β* = 0.406, 95% CI = [0.385, 0.427], *p* < 0.001). No significant association was observed between online healthcare services utilization and depressive symptoms (*β* = −0.015, 95% CI = [−0.092, 0.061], *p* = 0.697). When all three variables were included in the adjusted model, the previously significant association between eHealth literacy and depressive symptoms became nonsignificant (*β* = −0.002, 95% CI = [−0.024, 0.019], *p* = 0.827).

**Figure 1:**
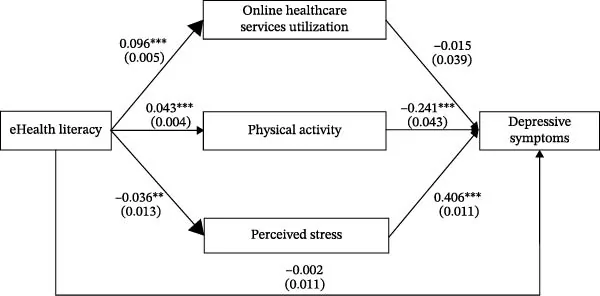
Mediating effects of online healthcare services utilization, physical activity, and perceived stress. The coefficients of ordinary least squares regression represented marginal effects, with robust standard errors in parentheses.  ^∗^ *p* < 0.05,  ^∗∗^ *p* < 0.01,  ^∗∗∗^ *p* < 0.001.

Bootstrap mediation analysis with 5000 resamples was conducted to quantify the indirect correlational associations between eHealth literacy and depressive symptoms, with all pathway estimates summarized in Table 3. The total association between eHealth literacy and depressive symptoms was statistically significant (*β* = −0.029, 95% CI = [−0.051, −0.007], *p* = 0.010), whereas the direct association between the two variables was nonsignificant (*β* = −0.002, 95% CI = [−0.023, 0.019], *p* = 0.827).

**Table 3:** Results of the bootstrap mediation effect test.

Potential pathways	*β*	95% CI	*p*
Direct effect (eHealth literacy)	−0.002	[−0.023, 0.019]	0.827
Indirect effect	−0.027	[−0.025, −0.009]	<0.001
Online healthcare services utilization	−0.002	[−0.009, 0.006]	0.697
Physical activity	−0.010	[−0.015, −0.006]	<0.001
Perceived stress	−0.015	[−0.040, −0.013]	0.005
Total effect	−0.029	[−0.051, −0.007]	0.010

*Note:* Control variables included age, gender, residence, educational attainment, marital status, activity of daily living (ADL) score, surviving children number, log‐transformed personal annual income, household Internet access, smartphone ownership, parental care responsibilities, and empty‐nest status.

Regarding specific indirect pathways, the correlational linkage mediated by reported physical activity frequency was statistically significant (*β* = −0.010, 95% CI = [−0.015, −0.006], *p* < 0.001), supporting Hypothesis 3. Similarly, perceived stress exhibited a significant indirect correlational association (*β* = −0.015, 95% CI = [−0.040, −0.013], *p* = 0.005), which supported Hypothesis 4. In contrast, the indirect pathway via online healthcare services utilization did not reach statistical significance (*β* = −0.002, 95% CI = [−0.009, 0.006], *p* = 0.697). This finding indicates that Hypothesis 2 was not statistically supported.

Overall, the aggregated indirect association across all candidate mediators was significant (*β* = −0.027, 95% CI = [−0.025, −0.009], *p* < 0.001), indicating a robust compound correlational pathway linking eHealth literacy to depressive symptoms through the proposed mediating variables.

### 4.4. Income‐Stratified Subgroup Analysis

As shown in Figure 2 and Table S6, the association between eHealth literacy and depressive symptoms differed across income subgroups. No statistically significant association was identified among low‐ income participants (*β* = −0.011, 95% CI = [−0.044, 0.022], *p* = 0.528). A significant negative association was observed for middle‐ income respondents (*β* = −0.041, 95% CI = [−0.078, −0.004], *p* = 0.028), and this negative association was stronger among high‐ income participants (*β* = −0.153, 95% CI = [−0.220, −0.086], *p* < 0.001). Results from the interaction model are displayed in Table S7. Consistent with these stratified results, the interaction between eHealth literacy and middle income was significantly negative (*β* = −0.054, 95% CI = [−0.101, −0.007], *p* = 0.026), as was the interaction between eHealth literacy and high income (*β* = −0.159, 95% CI = [−0.232, −0.085], *p* < 0.001), indicating that income moderated the association between eHealth literacy and depressive symptoms.

**Figure 2:**
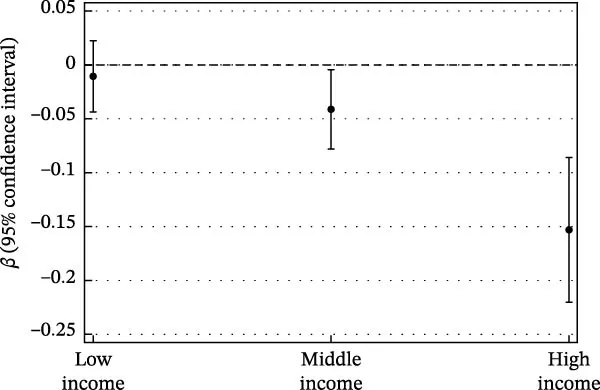
Income differences in the association between eHealth literacy and depressive symptoms.

### 4.5. Robustness Test

PSM was further applied to mitigate potential selection bias and unbalanced confounding characteristics in this observational study. Participants were divided into high and low eHealth literacy subgroups based on the sample mean of eHEALS scores, including 1684 participants (39.31%) in the high‐literacy group and 2600 participants (60.69%) in the low‐literacy group. All baseline covariates were incorporated as matching variables to balance the intergroup characteristics. As illustrated in Table S8, significant intergroup differences in multiple demographic and socioeconomic covariates existed before matching, whereas an adequate covariate balance was fully achieved after PSM implementation. Convergent results shown in Table S9 from nearest neighbor matching (*β* = −0.603, 95% CI = [−0.845, −0.360], *p* < 0.001), radius matching (*β* = −0.603, 95% CI = [−0.874, −0.331], *p* < 0.001) and kernel matching (*β* = −0.603, 95% CI = [−0.850, −0.355], *p* < 0.001) consistently confirmed the significant negative linkage between eHealth literacy and depressive symptoms, solidifying the reliability of the baseline findings.

IPW was finally conducted as an additional weighting‐based robustness test to further reduce the measured covariate imbalance. Consistent with the PSM grouping criteria, respondents were stratified into high and low eHealth literacy subgroups, and stable analytical weights were calculated based on the propensity score model adjusted for all covariates. The covariate balance summary presented in Table S10 further verified the effectiveness of the IPW adjustment: standardized mean differences of all covariates were close to zero after weighting, and the variance ratios were approximately equal to one, indicating excellent post‐weighting covariate balance. The IPW analytical results revealed that high eHealth literacy was associated with fewer depressive symptoms (ATE = −0.583, 95% CI = [−0.837, −0.329], *p* < 0.001).

## 5. Discussion

Drawing on the stress and coping theory, this study comprehensively examined the association between eHealth literacy and depressive symptoms among adults aged 50 years and older and further tested the mediating roles and explored income‐based heterogeneity. Results showed that compared with the low group of eHealth literacy, both the middle and high eHealth literacy groups had lower depressive symptoms, indicating a significant negative correlation between eHealth literacy and depressive symptoms among adults aged 50 years and older. These findings are consistent with recent cross‐cultural and regional investigations from the EU, Japan, and South Korea [52, 53]. Such concordant results suggest that higher eHealth literacy was inversely associated with depressive symptoms across the cultural contexts examined in previous studies.

The bootstrap analysis revealed that self‐reported physical activity frequency and perceived stress constituted statistically significant indirect pathways, whereas the indirect association through online healthcare service utilization was not statistically significant. These results align with and extend the stress and coping theory, as adults aged 50 years and older are exposed to a plethora of stressors throughout the aging process. This vulnerability renders them highly susceptible to the development of depressive symptoms. Here, eHealth literacy appears to be a linchpin in the observed association with depressive symptoms among this demographic. Our findings suggest two potential pathways that may help explain the observed inverse association between eHealth literacy and depressive symptoms. First, higher eHealth literacy may be associated with a better understanding of and greater engagement in healthy lifestyles among adults aged 50 years and older. By fostering a comprehensive recognition of the profound advantage of physical activity on both somatic and psychological well‐being, it may be associated with more frequent engagement in physical activity [54]. Physical activity may not only fortify physical constitution but also stimulate the cerebral release of endorphins. This biochemical response elicits a sense of well‐being, which may, in turn, contribute to the alleviation of depressive symptoms [18, 52].

Second, higher eHealth literacy was associated with lower perceived stress, which was, in turn, associated with fewer depressive symptoms. One possible explanation is that adults aged 50 years and older with higher eHealth literacy may be better able to access and evaluate health‐related information [11], supporting their appraisal of health‐related stressors and selection of appropriate coping strategies [55, 56]. This pattern is consistent with stress and coping theory, which emphasizes the roles of stress appraisal and perceived coping capacity. It is also consistent with cognitive vulnerability theories of depression, which emphasize the associations of persistent negative appraisal, reduced perceived control, and maladaptive cognitive patterns with depressive symptoms [23, 43, 44]. Thus, perceived stress may represent a statistical indirect pathway in the association between eHealth literacy and depressive symptoms. Nevertheless, the cross‐sectional data do not establish the temporal or causal ordering of these relationships.

The nonsignificant finding regarding online healthcare services warrants particular attention. Although higher eHealth literacy was associated with greater engagement with online health resources, such utilization bears no significant inverse association with depressive symptoms. One possible explanation is that online healthcare utilization alone may not necessarily translate into psychological benefits among older adults because the mental health value of digital health services may depend on service accessibility, usability, and users’ ability to effectively engage with online platforms. In practice, one critical barrier lies in age‐inappropriate digital platform design, consistent with prior appeals for senior‐friendly health interfaces proposed in cross‐cultural studies [57, 58]. A prior investigation revealed that even older adults with high eHealth literacy encounter barriers to identifying credible online health information, a constraint that ultimately diminishes the mental well‐being benefits of digital health utilization [28, 59].

The inverse association between eHealth literacy and depressive symptoms differed by income level. Stratified regression revealed no significant association within the low‐income group. Significant negative associations were observed in both the middle‐income and high‐income groups. Consistent with these stratified findings, significant interaction terms between eHealth literacy and middle income, as well as between eHealth literacy and high income, indicated that income moderated the inverse association between eHealth literacy and depressive symptoms, relative to the low‐income reference group. This disparity may stem from multifaceted resource constraints characteristic of low‐income settings. Barriers, such as inadequate internet access and limited availability of digital devices, likely hinder effective engagement with online health information. Furthermore, individuals with lower educational attainment may face difficulties in evaluating information credibility and frequently exhibit skepticism toward authoritative channels, which may further compromise the functional utility of eHealth literacy. Therefore, strengthening the potential contribution of eHealth literacy to mental health support for income‐level populations requires in‐depth research into their specific needs, usage barriers, and psychological mechanisms regarding eHealth services. Tailored interventions should be implemented to address the resource limitations and socioeconomic obstacles faced by lower income group, enabling them to benefit from the dividends of digital health development and more effectively support the mental health needs [7, 60].

The primary novelty of the present study resides in its problem‐driven analytical framework grounded in the Stress and Coping Theory to examine potential pathways linking eHealth literacy to depressive symptoms among adults aged 50 years and older. Unlike previous cross‐sectional studies that only confirmed a direct correlation between eHealth literacy and mental health outcomes. This study provides empirical evidence supporting two potential pathways: a cognitive‐emotional indirect pathway mediated by perceived stress and a single problem‐focused behavioral coping pathway represented by physical activity. By systematically clarifying these dual cognitive‐emotional and behavioral pathways that may help explain the inverse association between eHealth literacy and depressive symptoms, this study provides a theoretical basis for future longitudinal and intervention studies for middle‐aged and older adults.

This study has several strengths. It used a large sample of adults aged 50 years and older with broad geographic coverage across China and employed established instruments to assess eHealth literacy, depressive symptoms, and perceived stress. By examining multiple statistical indirect pathways and income‐stratified patterns alongside the primary association, this study adds new evidence on the relationship between eHealth literacy and depressive symptoms in this population. From a clinical and public health perspective, the findings suggest that eHealth literacy may be relevant when assessing the digital health information needs of individuals who report depressive symptoms. Healthcare providers and community health programs could consider individuals’ ability to locate, evaluate, and apply online health information when developing digital health education and mental health support services. These implications should be interpreted cautiously given the cross‐sectional design.

This study has several limitations. First, given its reliance on cross‐sectional survey data, establishing a definitive causal relationship between eHealth literacy and depressive symptoms remains beyond reach. Second, the measurement of online healthcare service utilization was constrained by data availability. Future research would benefit from incorporating multidimensional assessments of digital health service utilization. Third, physical activity was measured using a single self‐reported frequency item that did not capture activity duration, intensity, type, or energy expenditure. Therefore, the statistical indirect pathway involving self‐reported physical activity frequency should be interpreted cautiously. Fourth, limited potential intermediate variables were examined, such as online healthcare service utilization, physical activity, and perceived stress. Future research should incorporate additional potential intermediate constructs, particularly psychological capital (e.g., self‐efficacy and resilience) and social capital (e.g., social participation and network support), which may represent potential pathways linking eHealth literacy to mental health outcomes, thereby warranting inclusion in subsequent investigations. Fifth, the study sample was restricted to adults aged 50 years and older. Findings may not be generalizable to younger middle‐aged adults (e.g., those aged 45–49). Future studies with broader age coverage are needed to examine whether the observed associations hold across the full middle‐aged and older adult population.

## 6. Conclusion

This cross‐sectional study found a significant inverse association between eHealth literacy and depressive symptoms among adults aged 50 years and older. Statistically significant indirect associations were observed through self‐reported physical activity frequency and perceived stress; however, the cross‐sectional data do not establish the temporal or causal ordering of these relationships. Income‐stratified analyses suggested that the inverse association was more evident among participants in the middle‐ and high‐income groups. These findings indicate that eHealth literacy may be relevant when developing future mental health support strategies for adults aged 50 years and older. Longitudinal and intervention studies are needed to determine whether changes in eHealth literacy are followed by subsequent changes in depressive symptoms.

## Funding

This study was supported by the National Natural Science Foundation of China (Grants 72504278 and 72404274), the Fundamental Research Funds for the Central Universities, the Research Funds of Renmin University of China (Grants 24XNN083 and 25XNKJ08), the Key Project of the National Social Science Fund (Grant 24&ZD167).

## Disclosure

The present study is a cross‐sectional investigation. We confirm that the manuscript has been prepared in compliance with the STROBE Statement for cross‐sectional studies, and all recommended items have been appropriately addressed.

## Ethics Statement

The survey protocol was reviewed and approved by the Ethics Committee of Renmin University of China before data collection. Under the institution’s ethics review procedures in place at the time, questionnaire‐based social science studies that did not involve the collection of biological specimens or other biomedical materials were not assigned a formal ethics approval number. Accordingly, no formal approval number was issued for this survey. All participants provided written informed consent before participating in the survey, and the data used in the present analysis were anonymized.

## Conflicts of Interest

The authors declare no conflicts of interest.

## Supporting Information

Additional supporting information can be found online in the Supporting Information section.

## Supporting information


**Supporting Information** Supporting Information tables (Tables S1–S10) report measurement properties from principal component analysis and confirmatory factor analysis, path analysis outputs, results from robustness tests, and balance tests for propensity score matching and inverse probability weighting related to eHealth literacy and depressive symptoms.

## Data Availability

The data that support the findings of this study are available from the corresponding author upon reasonable request. The data are not publicly available due to privacy and ethical restrictions.
