# Supplementary material for: eHealth Literacy and Depressive Symptoms Among Adults Aged 50 Years and Older in China: A National Cross‐Sectional Study
Source: Depress Anxiety. 2026 Oct 28;2026:3142110. doi: 10.1155/da/3142110 (PMC13714633; doi:10.1155/da/3142110)
Supplement: Supplementary file 1 [file DA-2026-3142110-s001.docx]

**Supplementary material**

**Table S1.** Factor loading and uniqueness variances of principal component analysis.

| Item | Factor 1 Loading | Factor 2 Loading | Uniqueness |
| --- | --- | --- | --- |
| Negative-item-1 | 0.001 | 0.675 | 0.545 |
| Negative-item-2 | 0.022 | 0.608 | 0.630 |
| Negative-item-3 | −0.175 | 0.512 | 0.708 |
| Positive-item-1 | 0.788 | −0.014 | 0.380 |
| Positive-item-2 | 0.746 | 0.104 | 0.432 |
| Negative-item-4 | 0.088 | 0.615 | 0.614 |
| Positive-item-3 | 0.715 | 0.019 | 0.488 |
| Positive-item-4 | 0.779 | −0.025 | 0.393 |
| Negative-item-5 | 0.078 | 0.593 | 0.643 |
| Negative-item-6 | −0.153 | 0.558 | 0.665 |

**Table S2.** Fit indices of two-factor confirmatory factor analysis models.

| **Latent construct** | **Standardized factor loading range** | **CFI** | **TLI** | **SRMR** | **RMSEA** |
| --- | --- | --- | --- | --- | --- |
| Positive-item | 0.596–0.707 | 0.999 | 0.997 | 0.006 | 0.020 |
| Negative-item | 0.393–0.578 | 0.955 | 0.925 | 0.028 | 0.050 |

*Notes:* CFI = Comparative Fit Index (cutoff > 0.90); TLI = Tucker-Lewis Index (cutoff > 0.90); SRMR = Standardized Root Mean Squared Residual (cutoff < 0.05); RMSEA = Root Mean Squared Error of Approximation (cutoff < 0.05).

**Table S3.** The association between different levels of eHealth literacy and depressive symptoms.

| **Variables** | ***β*** | **95%*CI*** | ***P*** |
| --- | --- | --- | --- |
| eHealth literacy (ref: low group) |  |  |  |
| Middle group | -0.345 | [-0.634, -0.055] | 0.020 |
| High group | -0.759 | [-1.051, -0.467] | <0.001 |
| Age | 0.030 | [0.009, 0.052] | 0.006 |
| Female | 0.475 | [0.239, 0.712] | <0.001 |
| Residence (ref: rural) | -0.690 | [-0.951, -0.430] | <0.001 |
| Education level (ref: primary school and below) |  |  |  |
| Middle school | -0.044 | [-0.367, 0.279] | 0.791 |
| High school | 0.047 | [-0.278, 0.371] | 0.778 |
| College and above | -0.175 | [-0.669, 0.318] | 0.485 |
| Marital status (ref: no) | -0.975 | [-1.664, -0.285] | 0.006 |
| Activity of daily living | 0.900 | [0.628, 1.171] | <0.001 |
| Household Internet access (ref: no) | -1.016 | [-1.534, -0.497] | <0.001 |
| Smartphone ownership (ref: no) | -0.656 | [-1.782, 0.469] | 0.253 |
| Number of surviving children | 0.325 | [0.146, 0.503] | <0.001 |
| Parental care responsibilities (ref: no) | 0.216 | [-0.091, 0.524] | 0.168 |
| Empty-nest status (ref: no) | 0.582 | [-0.215, 1.379] | 0.153 |
| Log(income) | 0.397 | [0.167, 0.628] | 0.001 |
| Constant | 2.924 | [0.388, 5.459] | 0.024 |

*Notes: N=4,284, R^2^=0.053. Ref: reference; CI: confidence interval.*

**Table S4.** Associations between eHealth literacy and mediating variables.

| **Variables** | **Online healthcare services utilization** | | | **Physical activity** | | | **Perceived stress** | | |
| --- | --- | --- | --- | --- | --- | --- | --- | --- | --- |
|  | ***β*** | **95%*CI*** | ***P*** | ***β*** | **95%*CI*** | ***P*** | ***β*** | **95%*CI*** | ***P*** |
| eHealth literacy | 0.096 | [0.087, 0.106] | <0.001 | 0.043 | [0.035, 0.052] | <0.001 | −0.036 | [−0.062, −0.011] | 0.005 |
| Age | 0.007 | [-0.001, 0.015] | 0.072 | 0.018 | [0.011, 0.025] | <0.001 | 0.027 | [0.005, 0.049] | 0.017 |
| Female | 0.019 | [-0.070, 0.108] | 0.673 | −0.103 | [−0.184, −0.022] | 0.013 | 0.631 | [0.354, 0.907] | <0.001 |
| Residence (ref: rural) | 0.100 | [0.002, 0.197] | 0.046 | 0.159 | [0.068, 0.249] | 0.001 | −0.552 | [−0.846, −0.257] | <0.001 |
| Education level (ref: primary school and below) |  |  |  |  |  |  |  |  |  |
| Middle school | 0.229 | [0.098, 0.360] | 0.001 | −0.151 | [−0.264, −0.037] | 0.009 | 0.068 | [−0.308, 0.444] | 0.723 |
| High school | 0.325 | [0.198, 0.452] | <0.001 | −0.007 | [−0.118, 0.104] | 0.905 | −0.144 | [−0.533, 0.246] | 0.470 |
| College and above | 0.528 | [0.338, 0.717] | <0.001 | 0.223 | [0.068, 0.378] | 0.005 | −0.712 | [−1.321, −0.103] | 0.022 |
| Marital status (ref: no) | -0.142 | [-0.393, 0.109] | 0.267 | 0.013 | [−0.255, 0.281] | 0.923 | 0.008 | [−0.704, 0.721] | 0.982 |
| Activity of daily living | -0.053 | [-0.149, 0.043] | 0.277 | −0.210 | [−0.295, −0.124] | <0.001 | 0.680 | [0.471, 0.888] | <0.001 |
| Household Internet access (ref: no) | 0.309 | [0.096, 0.521] | 0.004 | 0.323 | [0.112, 0.534] | 0.003 | −1.461 | [−2.024, −0.899] | <0.001 |
| Smartphone ownership (ref: no) | 0.381 | [-0.008, 0.771] | 0.055 | 0.035 | [−0.249, 0.318] | 0.811 | −1.726 | [−2.694, −0.758] | <0.001 |
| Number of surviving children | 0.033 | [-0.032, 0.098] | 0.319 | 0.057 | [−0.003, 0.118] | 0.064 | 0.345 | [0.148, 0.543] | 0.001 |
| Parental care responsibilities (ref: no) | 0.224 | [0.116, 0.331] | <0.001 | 0.055 | [−0.046, 0.155] | 0.286 | 0.101 | [−0.264, 0.467] | 0.586 |
| Empty-nest status (ref: no) | 0.043 | [-0.255, 0.342] | 0.776 | −0.197 | [−0.507, 0.112] | 0.211 | 0.788 | [−0.023, 1.598] | 0.057 |
| Log(income) | 0.242 | [0.158, 0.327] | <0.001 | 0.153 | [0.074, 0.232] | <0.001 | −0.350 | [−0.617, −0.083] | 0.010 |
| Constant | 3.902 | [2.902, 4.902] | <0.001 | 2.805 | [1.977, 3.632] | <0.001 | 13.820 | [11.337, 16.304] | <0.001 |

*Notes: N=4,284,* $R_{Online healthcare services utilization}^{2}$*=0.164,* $R_{Physical activity}^{2}$*=0.065,* $R_{Perceived stress}^{2}$*=0.047. Ref: reference; CI: confidence interval.*

**Table S5.** OLS regression results for associations among eHealth literacy, mediating variables and depressive symptoms.

| **Variables** | ***β*** | ***95% CI*** | ***P*** |
| --- | --- | --- | --- |
| eHealth literacy | -0.002 | [-0.024, 0.019] | 0.827 |
| Online healthcare services utilization | -0.015 | [-0.092, 0.061] | 0.697 |
| Physical activity | -0.241 | [-0.325, -0.157] | <0.001 |
| Perceived stress | 0.406 | [0.385, 0.427] | <0.001 |
| Age | 0.020 | [0.001, 0.039] | 0.043 |
| Female | 0.200 | [-0.008, 0.408] | 0.059 |
| Residence (ref: rural) | -0.417 | [-0.645, -0.190] | <0.001 |
| Education level (ref: primary school and below) |  |  |  |
| Middle school | -0.102 | [-0.393, 0.189] | 0.491 |
| High school | 0.072 | [-0.216, 0.360] | 0.624 |
| College and above | 0.168 | [-0.244, 0.580] | 0.425 |
| Marital status (ref: no) | -1.014 | [-1.633, -0.395] | 0.001 |
| Activity of daily living | 0.575 | [0.314, 0.837] | <0.001 |
| Household Internet access (ref: no) | -0.347 | [-0.852, 0.157] | 0.177 |
| Smartphone ownership (ref: no) | 0.086 | [-0.946, 1.118] | 0.871 |
| Number of surviving children | 0.200 | [0.042, 0.358] | 0.013 |
| Parental care responsibilities (ref: no) | 0.178 | [-0.088, 0.445] | 0.190 |
| Empty-nest status (ref: no) | 0.180 | [-0.566, 0.926] | 0.636 |
| Income level (ref: Low income) | 0.563 | [0.360, 0.766] | <0.001 |
| Middle income | -1.217 | [-3.644, 1.209] | 0.325 |
| High income | -0.002 | [-0.024, 0.019] | 0.827 |
| Constant | -0.015 | [-0.092, 0.061] | 0.697 |

*Notes: N=4,284, R^2^=0.276. Ref: reference; CI: confidence interval.*

**Table S6.** Income differences in the association between eHealth literacy and depressive symptoms.

| **Variables** | **Low income**  **(N=1,549)** | | | **Middle income**  **(N=1,562)** | | | **High income**  **(N=1,173)** | | |
| --- | --- | --- | --- | --- | --- | --- | --- | --- | --- |
|  | ***β*** | **95%*CI*** | ***P*** | ***β*** | **95%*CI*** | ***P*** | ***β*** | **95%*CI*** | ***P*** |
| eHealth literacy | -0.011 | [-0.044, 0.022] | 0.528 | -0.041 | [-0.078, -0.004] | 0.028 | -0.153 | [-0.220, -0.086] | <0.001 |
| Age | -0.001 | [-0.033, 0.031] | 0.938 | 0.014 | [-0.020, 0.048] | 0.419 | 0.100 | [0.033, 0.167] | 0.003 |
| Female | 0.728 | [0.321, 1.134] | <0.001 | 0.003 | [-0.382, 0.387] | 0.989 | 0.750 | [0.295, 1.206] | 0.001 |
| Residence (ref: rural) | -1.051 | [-1.490, -0.612] | <0.001 | -0.868 | [-1.347, -0.389] | <0.001 | -0.136 | [-0.625, 0.353] | 0.586 |
| Education level (ref: primary school and below) |  |  |  |  |  |  |  |  |  |
| Middle school | 0.319 | [-0.193, 0.832] | 0.222 | -0.086 | [-0.662, 0.490] | 0.770 | -1.335 | [-1.998, -0.671] | <0.001 |
| High school | 0.191 | [-0.376, 0.757] | 0.509 | -0.212 | [-0.770, 0.345] | 0.455 | -0.339 | [-0.951, 0.273] | 0.278 |
| College and above | -0.154 | [-1.266, 0.958] | 0.786 | -0.194 | [-1.006, 0.618] | 0.639 | -0.683 | [-1.470, 0.105] | 0.089 |
| Marital status (ref: no) | -1.340 | [-2.294, -0.387] | 0.006 | -0.896 | [-2.159, 0.367] | 0.164 | -0.845 | [-2.479, 0.789] | 0.311 |
| Activity of daily living | 1.273 | [0.800, 1.746] | <0.001 | 0.688 | [0.290, 1.086] | 0.001 | 0.763 | [0.268, 1.257] | 0.003 |
| Household Internet access (ref: no) | -0.963 | [-1.695, -0.230] | 0.010 | -0.260 | [-1.308, 0.788] | 0.627 | -1.806 | [-2.835, -0.778] | 0.001 |
| Smartphone ownership (ref: no) | -1.657 | [-3.432, 0.118] | 0.067 | -0.357 | [-2.498, 1.784] | 0.744 | 1.082 | [-0.455, 2.618] | 0.167 |
| Number of surviving children | 0.391 | [0.087, 0.695] | 0.012 | 0.412 | [0.112, 0.711] | 0.007 | 0.200 | [-0.124, 0.524] | 0.225 |
| Parental care responsibilities (ref: no) | 0.001 | [-0.579, 0.581] | 0.997 | -0.042 | [-0.532, 0.447] | 0.866 | 0.465 | [-0.086, 1.016] | 0.098 |
| Empty-nest status (ref: no) | 0.168 | [-1.001, 1.337] | 0.778 | 0.545 | [-0.809, 1.900] | 0.430 | 1.969 | [0.235, 3.704] | 0.026 |
| Log(income) | 3.281 | [-1.101, 7.662] | 0.142 | 8.264 | [3.337, 13.192] | 0.001 | 3.349 | [-2.598, 9.296] | 0.269 |
| Constant | -0.011 | [-0.044, 0.022] | 0.528 | -0.041 | [-0.078, -0.004] | 0.028 | -0.153 | [-0.220, -0.086] | <0.001 |

*Notes:*$R_{Low income}^{2}$*=0.075,* $R_{Middle income}^{2}$*=0.047,* $R_{High income}^{2}$*=0.099. Ref: reference; CI: confidence interval*

**Table S7.** Adjusted associations of eHealth literacy, income level, and their interactive terms with depressive symptoms.

| **Variables** | ***β*** | ***95% CI*** | ***P*** |
| --- | --- | --- | --- |
| eHealth literacy | 0.013 | [-0.018, 0.045] | 0.412 |
| eHealth literacy × Middle income | -0.054 | [-0.101, -0.007] | 0.026 |
| eHealth literacy × High income | -0.159 | [-0.232, -0.085] | <0.001 |
| Age | 0.024 | [0.003, 0.045] | 0.026 |
| Female | 0.442 | [0.203, 0.681] | <0.001 |
| Residence (ref: rural) | -0.642 | [-0.904, -0.379] | <0.001 |
| Education level (ref: primary school and below) |  |  |  |
| Middle school | -0.098 | [-0.427, 0.230] | 0.558 |
| High school | 0.008 | [-0.320, 0.337] | 0.961 |
| College and above | -0.147 | [-0.640, 0.346] | 0.559 |
| Marital status (ref: no) | -0.956 | [-1.644, -0.267] | 0.007 |
| Activity of daily living | 0.915 | [0.643, 1.188] | <0.001 |
| Household Internet access (ref: no) | -0.981 | [-1.505, -0.457] | <0.001 |
| Smartphone ownership (ref: no) | -0.581 | [-1.715, 0.553] | 0.315 |
| Number of surviving children | 0.310 | [0.132, 0.488] | 0.001 |
| Parental care responsibilities (ref: no) | 0.186 | [-0.124, 0.495] | 0.239 |
| Empty-nest status (ref: no) | 0.651 | [-0.149, 1.451] | 0.111 |
| Income level (ref: Low income) |  |  |  |
| Middle income | 1.500 | [0.197, 2.803] | 0.024 |
| High income | 4.668 | [2.616, 6.719] | <0.001 |
| Constant | 3.113 | [0.539, 5.686] | 0.018 |

*Notes: N=4,284, R^2^=0.053. Ref: reference; CI: confidence interval.*

**Table S8.** The reduction of errors of variables before and after matching.

| **Variables** | **Unmatched /**  **Matched** | **SD** | | **bias**  **(%)** | **reduced**  **\|bias\|(%)** | ***t*-Test** | |
| --- | --- | --- | --- | --- | --- | --- | --- |
|  |  | **Treated** | **Control** |  |  | ***t*** | ***P*** |
| Age | Unmatched | 58.225 | 60.200 | -28.2 | 93.8 | -9.15 | <0.001 |
|  | Matched | 58.221 | 58.098 | 1.7 |  | 0.68 | 0.497 |
| Female (ref: male) | Unmatched | 0.486 | 0.527 | -8.2 | 74.6 | -2.62 | 0.009 |
|  | Matched | 0.486 | 0.496 | -2.1 |  | -0.375 | 0.454 |
| Residence (ref: rural) | Unmatched | 0.692 | 0.685 | 1.4 | 19.5 | 0.46 | 0.646 |
|  | Matched | 0.692 | 0.697 | -1.2 |  | -0.42 | 0.676 |
| Middle school (ref: primary school and below) | Unmatched | 0.338 | 0.356 | -3.8 | 42.0 | -1.21 | 0.226 |
|  | Matched | 0.338 | 0.349 | -2.2 |  | -0.79 | 0.428 |
| High school (ref: primary school and below) | Unmatched | 0.388 | 0.286 | 21.7 | 99.4 | 6.87 | <0.001 |
|  | Matched | 0.388 | 0.387 | 0.1 |  | 0.04 | 0.967 |
| College and above (ref: primary school and below) | Unmatched | 0.084 | 0.080 | 1.6 | 80.8 | 0.50 | 0.619 |
|  | Matched | 0.084 | 0.083 | 0.3 |  | 0.11 | 0.915 |
| Marital status (ref: no) | Unmatched | 0.951 | 0.893 | 21.8 | 87.7 | 7.24 | <0.001 |
|  | Matched | 0.951 | 0.959 | -2.7 |  | -1.25 | 0.213 |
| Activity of daily living | Unmatched | 5.094 | 5.128 | -6.4 | 76.5 | -2.08 | 0.038 |
|  | Matched | 5.094 | 5.086 | 1.5 |  | 0.63 | 0.529 |
| Household Internet access (ref: no) | Unmatched | 0.958 | 0.941 | 7.9 | 79.8 | 2.57 | 0.010 |
|  | Matched | 0.958 | 0.962 | -1.6 |  | -0.64 | 0.521 |
| Smartphone ownership (ref: no) | Unmatched | 0.987 | 0.989 | -1.9 | -21.1 | -0.59 | 0.555 |
|  | Matched | 0.987 | 0.990 | -2.3 |  | 0.82 | 0.412 |
| Surviving children number | Unmatched | 1.504 | 1.477 | 3.8 | -4.4 | 1.21 | 0.225 |
|  | Matched | 1.504 | 1.475 | 3.9 |  | 1.45 | 0.147 |
| Parental care responsibilities (ref: no) | Unmatched | 0.216 | 0.151 | 16.9 | 98.3 | 5.33 | <0.001 |
|  | Matched | 0.216 | 0.214 | 0.3 |  | 0.10 | 0.924 |
| Empty-nest status (ref: no) | Unmatched | 0.041 | 0.067 | -11.4 | 70.0 | -3.75 | <0.001 |
|  | Matched | 0.041 | 0.033 | 3.4 |  | 1.47 | 0.140 |
| Log(income) | Unmatched | 1.982 | 1.737 | 40.1 | 98.1 | -9.15 | <0.001 |
|  | Matched | 1.982 | 1.978 | 0.7 |  | 0.28 | 0.783 |

**Table S9.** Results of propensity score matching.

| **Matching method** | ***β*** | ***95% CI*** | ***P*** |
| --- | --- | --- | --- |
| Nearest neighbor matching | -0.603 | [-0.845, -0.360] | <0.001 |
| Radius matching | -0.603 | [-0.874, -0.331] | <0.001 |
| Kernel matching | -0.603 | [-0.850, -0.355] | <0.001 |

**Table S10.** Covariates balance before and after inverse probability weighting.

| Covariates | Standardized Mean Difference (Raw) | Standardized Mean Difference (Weighted) | Variance Ratio (Raw) | Variance Ratio (Weighted) |
| --- | --- | --- | --- | --- |
| Age | -0.282 | 0.002 | 0.755 | 0.928 |
| Gender | 0.082 | -0.001 | 1.002 | 1.000 |
| Residence | 0.014 | 0.008 | 0.988 | 0.993 |
| Educational attainment (ref: primary school and below) |  |  |  |  |
| Middle school | -0.038 | -0.003 | 0.976 | 0.998 |
| High school | 0.217 | 0.004 | 1.162 | 1.002 |
| College and above | 0.016 | 0.004 | 1.049 | 1.013 |
| Marital status | 0.218 | 0.008 | 0.487 | 0.973 |
| Activity of daily living | -0.064 | -0.001 | 0.677 | 1.042 |
| Household Internet access | 0.079 | 0.004 | 0.719 | 0.983 |
| Smartphone ownership | -0.019 | 0.006 | 1.185 | 0.952 |
| Number of surviving children | 0.038 | -0.009 | 0.892 | 0.922 |
| Parental care responsibilities | 0.169 | -0.013 | 1.323 | 0.980 |
| Empty-nest status | -0.114 | -0.010 | 0.630 | 0.960 |
| Log(income) | 0.401 | -0.004 | 0.892 | 0.913 |

*Notes:* Raw = Unweighted pre-IPW sample; Weighted = Weighted post-IPW sample. Standardized mean differences < 0.1 indicate acceptable covariate balance; variance ratios close to 1 suggest balanced variance distribution between groups. All covariates were included in the propensity score model for IPW weight calculation.
